# Novel MS vital sign: multi‐sensor captures upper and lower limb dysfunction

**DOI:** 10.1002/acn3.50988

**Published:** 2020-02-26

**Authors:** Alireza Akhbardeh, Jennifer K. Arjona, Kristen M. Krysko, Bardia Nourbakhsh, Pierre Antoine Gourraud, Jennifer S. Graves

**Affiliations:** ^1^ Johns Hopkins University School of Medicine Baltimore Maryland; ^2^ Department of Neurology UCSF Weill Institute for Neurosciences University of California San Francisco California; ^3^ Department of Neurology Johns Hopkins University School of Medicine Baltimore Maryland; ^4^ CHU INSERM Centre de Recherche en Transplantation et Immunologie Institut de Transplantation Urologie Néphrologie (ITUN) Nantes Université UMR 1064 ATIP‐Avenir Nantes France; ^5^ Department of Neurosciences University of California San Diego California

## Abstract

**Objective:**

To create a novel neurological vital sign and reliably capture MS‐related limb disability in less than 5 min.

**Methods:**

Consecutive patients meeting the 2010 MS diagnostic criteria and healthy controls were offered enrollment. Participants completed finger and foot taps wearing the MYO‐band© (accelerometer, gyroscope, and surface electromyogram sensors). Signal processing was performed to extract spatiotemporal features from raw sensor data. Intraclass correlation coefficients (ICC) assessed intertest reproducibility. Spearman correlation and multivariable regression methods compared extracted features to physician‐ and patient‐reported disability outcomes. Partial least squares regression identified the most informative extracted textural features.

**Results:**

Baseline data for 117 participants with MS (EDSS 1.0–7.0) and 30 healthy controls were analyzed. ICCs for final selected features ranged from 0.80 to 0.87. Time‐based features distinguished cases from controls (*P* = 0.002). The most informative combination of extracted features from all three sensors strongly correlated with physician EDSS (finger taps *r_s_* = 0.77, *P* < 0.0001; foot taps *r_s_* = 0.82, *P* < 0.0001) and had equally strong associations with patient‐reported outcomes (WHODAS, finger taps *r_s_* = 0.82, *P* < 0.0001; foot taps *r_s_* = 0.82, *P* < 0.0001). Associations remained with multivariable modeling adjusted for age and sex.

**Conclusions:**

Extracted features from the multi‐sensor demonstrate striking correlations with gold standard outcomes. Ideal for future generalizability, the assessments take only a few minutes, can be performed by nonclinical personnel, and wearing the band is nondisruptive to routine practice. This novel paradigm holds promise as a new neurological vital sign.

## Introduction

Capturing with precision the extent of MS‐related disability is critical for effective clinical care and the development of new outcome metrics for rapid testing of therapeutic agents. To date, outcomes research in MS has centered on periodic clinical exams, which may be insensitive to changes over the short term (the 1–2 years of early stage clinical trials) and only capture a single semiquantitative snapshot of the patient’s performance. Single limb progression, which may be critical to quality of life and for screening of neuroprotective agents in early stage trials, has not been used as a primary outcome in MS studies largely due to lack of reliable and accessible quantitative measures of limb function. With the advent of mass production of sensors in the gaming and computer control industry, there is an opportunity to extend the traditional neurological exam with biosensors already in use outside the realm of health applications.

While accelerometers have been the most commonly studied sensor in MS research to date,^1^ their use in combination with other sensors has been more limited and the focus of prior studies has been to garner an assessment of physical activity rather than progression of limb dysfunction. Accelerometers used in the context of generating step counts are also insensitive to upper limb dysfunction and activity levels of those with restricted ambulation. Gyroscopes have been used in MS to assess balance‐related function and rarely to improve the quality of accelerometer data.^1^ The recreational use of surface electromyogram (sEMG) sensors has entered the gaming and computer control industry, but use of the commercially available sensors in clinical applications has been limited. They offer the unique ability to noninvasively measure muscle electrical activity during exam tasks and provide quantitative assessments of limb movement.

Using a sophisticated signal processing approach, we sought to validate a wearable multi‐sensor device (MYO©) for detection of upper and lower limb dysfunction in MS patients. We determined if extracted features from the three sensors (surface electromyogram, gyroscope, and accelerometer), individually and in combination, can be reliably measured, are associated with standard and validated measures of MS disability, and to evaluate their potential to help detect subtle dysfunction in comparison to healthy controls.

## Materials and Methods

### Participants

Participants were recruited from the UCSF Adult MS clinic (age ≥ 18 years, May 2016 to February 2018). They were offered enrollment and testing was performed at the time of their routine clinic visits. Diagnosis of MS, as defined by the International 2010 revised McDonald criteria,^2^ was confirmed by clinical record and MRI review. Both relapsing and progressive subtypes of MS were enrolled, but patients had to be able to attempt finger and foot taps. We also enrolled a group of 30 healthy control subjects free of neurological disease or any physical impairment. This control group consisted of nonblood‐related family members and friends of patients with MS and volunteers from the UCSF community. The absence of neurologic symptoms or other physical limitations was confirmed by a brief intake questionnaire.

### Standard protocol approvals and informed consent

The Committee on Human Subjects at UCSF approved this study, which is in compliance with the principles of the Declaration of Helsinki. All MS and control participants completed written informed consent.

### Procedures

In this cross‐sectional study, before or after the clinical visit, MS subjects completed secure RedCap questionnaires on provided tablets for clinical and treatment history, and self‐reported disability metrics (digital‐Expanded Disability Status Scale (EDSS), World Health Organization Disability Assessment Schedule 2.0 (WHODAS 2.0), described below) with supervision from the study coordinator to ensure completion. During the office visit exam, the MYO‐band was placed at the widest point of the upper forearm during routine finger tap testing. Markers on the wristband ensured the device was worn in the same orientation for all participants. The participant was asked to complete 20 taps with the right forefinger and thumb as quickly as possible with demonstration by the examiner of 4‐inch amplitude and 4‐Hz taps. Then the device was moved to the left arm and measurements repeated. The device was then placed over the widest part of the upper right calf. The participant was instructed from a sitting position to leave the heel of the foot on the ground and quickly tap the ball of the foot 20 times with large amplitude. The examiner demonstrated 4‒6‐inch amplitude and 4‐Hz taps. Measurements were repeated for the left leg. Each tapping task was repeated twice.

During these assessments, accelerometer (ACC), gyroscope (GYR), and surface EMG (sEMG) data were collected from MYO through a Bluetooth connection to an encryption‐protected laptop with both immediate signal processing and postprocessing metrics extraction as described below. At the end of the clinical visit, physician‐derived EDSS scores were entered into a separate RedCap EDSS form.

### Clinical measurements

Patients were queried for handedness. Physician scored and patient‐reported disability measures were assessed including both physician‐derived EDSS and patient‐reported telephone EDSS score adapted for digital questionnaire use.^3^ For potentially more patient‐focused real‐life outcomes we used the WHODAS 2.0, which includes six domains of function: cognition, mobility, self‐care, interacting with other people, life activities, and participating in community activities.^4^

### Sensor data acquisition

Modification to the data acquisition software development kit (SDK, Thalmic labs) and additional proprietary C++ code was made to export all of the MYO sensor data to text files in ASCII format at varied frame rates as set by device defaults and firmware.

### Signal processing and metrics extraction

#### Time‐based analysis

**Figure 1:**
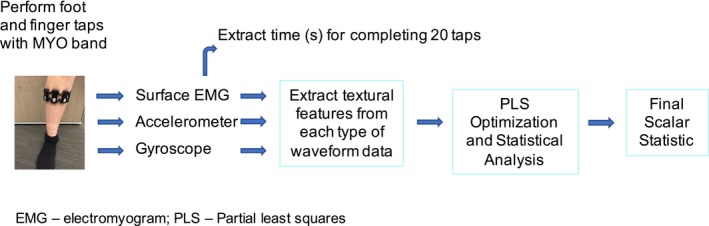
Overview of signal processing approach. EMG, electromyogram, PLS, Partial least squares. Participants performed 20 finger and foot taps as fast as they could while wearing the MYO‐band. The surface EMG data were used to extract the times for completing 20 taps for each limb. All three sensors were used for textural feature extraction. A PLS method was used to determine which sensor(s) and textural features were most informative. Lastly, a final scalar statistic for the textural analysis (“T‐metric”) was derived from the PLS model.

An overview of the signal processing approach is presented in Figure 1. The total duration of time needed to complete 20 taps was extracted from the sEMG sensor data. All eight channels of sEMG data were used. Raw data were converted to binary heatmaps using thresholding methods.^5^ Each heatmap was then smoothed using an imaging filter (moving average based) to remove motion artifacts and noise. Start and end times for completion of the 20 foot or finger taps were generated using an average of the start and end times for the individual EMG channels. Durations in seconds for all four limbs were generated.

#### Spatiotemporal texture‐based analysis

Textural features (shape, regularity of signal) of the waveform data from all three sensors (ACC, sEMG, and GYR) were extracted using well‐established and published image processing methods to more richly quantify performance during the tapping tasks.^6^, ^7^ We developed a data‐labeling algorithm to annotate peaks and extract tap events. This annotation algorithm employs a built‐in function of Matlab to find local maxima and peaks. Each individual finger or foot tap event was treated as a segment in the waveform of the sensor signal. Textural (“haralick_energy,” “haralick_correlation,” “haralick_contrast,” and “haralick_homogeneity”) and statistical features (“mean value for each tap,” “overall_energy,” and “overall_entropy”) were derived for each tap event (see Supplemental Methods Appendix A). Thus, for each set of 20 taps, there was ultimately an array of extracted textural features. The next critical step of identifying the most informative of these textural features is described below.

### Derivation of the most informative sensor metrics

The time‐based sensor‐derived metric described above measured the duration of time in seconds required for 20 taps in each limb. For the final texture‐based metric, to understand which textural features of the waveforms from the three sensors were more likely to be describing phenomena of interest (vs. noise) and best distinguished levels of function, we applied a partial least squares (PLS) method to all of the extracted features in 60% of the data (training dataset).^8^, ^9^ The gold standards (ground truth) for the PLS analyses were physician EDSS, patient‐reported EDSS, and the WHODAS score. The PLS‐based optimization returns the most important sensor(s) and textural features combination from the MYO for each limb in each participant. Then, standard statistical methods (mean, standard deviation, skewness, and kurtosis) are applied to the PLS signal to create a single, final summary statistic, a so‐called “T‐metric” (See Supplemental Methods Appendix B for mathematical modeling), representing all of the informative textural features predicting disability from the MYO for each limb in each participant. The final PLS model including the most informative textural features derived from the training data was then tested on the remaining 40% of participant data (replication dataset) to result in T‐metrics for each participant. The final T‐metrics from the replication data were compared to the MS outcome measures with Spearman correlation and regression models described below.

### Statistical analysis

For descriptive analysis, means, medians, or frequencies were presented as appropriate. Spearman (rho) correlations for nonparametric data were presented for comparison of sensor‐derived with patient‐reported and physician‐measured metrics. Handedness was addressed by creating a dominant limb category for metric results. Intraclass correlation coefficients (ICC) were used to assess reproducibility for repeated trials of both the time‐ and texture‐based metrics.

We compared case versus control differences in the time‐based sensor‐derived metrics with logistic regression, adjusted for age and sex. Extracted time‐ and texture‐based features were compared to patient‐reported and physician‐measured disability metrics with Spearman correlation coefficients. In addition, multivariable regression methods were used to assess the association of the sensor‐derived metrics with disability metrics (physician‐ and patient‐reported EDSS and WHODAS 2.0), adjusting for age and sex. To avoid over fitting and bias, PLS‐model training data were not used in any of the final analyses; only the replication dataset was used.

Matlab and STATA v12 were used for analyses. A two‐sided *P*‐value of less than 0.05 was considered significant.

### Data and material availability

Software may be available upon request to corresponding author and approval under nonexclusive use and nondisclosure contracts.

## Results

Clinical and sensor data were collected from 117 participants with MS and from 30 healthy controls. Descriptive comparisons of these groups are available in Table 1. Participants with MS were on average older than controls and predominantly female (Table 1), and these variables were evaluated for potential confounding effects for all multivariable analyses.

**Table 1:** Participant characteristics.

	MS (*n* = 117)	Controls (*n* = 30)
Mean age (SD)	47 (12.4)	39.7 (10.7)
Female sex *n* (%)	88 (75)	15 (50)
Median disease duration years (range)	10 (0.4–44)	–
Median EDSS (range)	2.5 (1–7)	–
Median self‐reported EDSS (range)	2 (0–7)	–
Median WHODAS total score (range)	13.2 (0–60.9)	–
Median 20‐finger tap duration s (range)		
Dominant hand	7.2 (2.4–19.6)	5.7 (3.6–10.2)
Nondominant hand	7.8 (2.3–19.9)	6.4 (4.0–8.2)
Median 20‐foot tap duration s (range)		
Dominant leg	8.2 (4.5–17.7)	6.4 (4.0–9.6)
Nondominant leg	8.6 (4.5–17.9)	6.5 (4.0–10.8)

MS, multiple sclerosis; EDSS, Expanded Disability Status Scale score; WHODAS, World Health Organization disability assessment score; s, seconds.

### Time‐based analyses

Surface electromyogram data were used to extract time‐based features for analysis. The total duration of time taken by participants to complete 20 finger or foot taps was calculated for all limbs using the sEMG data. Task duration times for dominant and nondominant limbs are presented in Table 1. In an unadjusted descriptive comparison, mean times were longer for MS cases for both upper and lower limbs. In a univariate regression analysis, age (per year) was associated with 0.07 sec longer 20‐finger tap duration time (95%CI 0.032, 0.11, *P* < 0.001) and 0.08 sec longer 20‐foot tap duration time (95%CI 0.038, 0.12, *P* < 0.001). Thus, age was included in all multivariable analyses below. Sex, however, was not associated with 20‐finger tap duration (*β* = −0.29, 95%CI −1.39, 0.80, *P* = 0.60) or 20‐foot tap duration (*β* = −0.025, 95%CI −1.27, 1.22, *P* = 0.967), but adjustment for sex was included for face validity.

In multivariable regression analysis adjusting for age and sex, participants with MS had 1.65 sec longer 20‐finger tap duration (95%CI 0.61, 2.69, *P* = 0.002) and 1.82 sec longer 20‐foot tap duration (95%CI 0.66, 2.97, *P* = 0.002) compared to healthy controls. Differences were still seen between cases and controls when the analysis was restricted to MS participants with no disability (EDSS < 2.0; 20‐foot tap duration *β* = 0.95 sec, 95%CI = 0.067, 1.84, *P* = 0.035).

Total duration combining times for the right and left limbs, and dominant or nondominant limbs were assessed for reproducibility and associations with MS outcomes. Test‒retest reliability was high for all MYO‐derived measures. The best test‒retest reliability was with the dominant limb in the upper extremity (ICC 0.87, Table 2).

**Table 2:** Intraclass correlation coefficients (ICC) of MYO‐band assessments.

Assessment	Combined right and left limbs	Dominant limb measure	Nondominant limb measure
Finger taps			
Time‐based analysis	0.76	0.87	0.70
Textural analysis	0.85	0.80	0.79
Foot taps			
Time‐based analysis	0.69	0.67	0.82
Textural analysis	0.81	0.71	0.71

The intertest repeatability is reported as intraclass correlation coefficients (ICC) for the different MYO‐band assessments. Time‐based analysis calculated total duration for completion of 20 tap movements for upper limb or lower limb, extracted from the surface EMG data. For the combined measure the total duration for right and left limbs was added together. The textural analysis leveraged data from all three sensors—accelerometer, gyroscope, and surface EMG (see Methods) and a partial least squares method determined the most informative features from the sensor data, which were used for the final ICC calculation.

Among participants with MS, physician exam‐derived EDSS scores (*r_s_* = 0.43, *P* = 1.2 × 10^−7^), patient self‐reported EDSS (*r_s_* = 0.48, *P* = 2.3 × 10^−9^), and WHODAS total scores (*r_s_* = 0.37, *P* = 7.2 × 10^−6^) were associated with 20‐finger tap duration of the dominant hand in Spearman correlation analyses (Table 3). As expected, stronger magnitude correlations were observed between 20‐foot tap durations and the outcomes measures, which are heavily weighted to ambulatory (lower extremity) function (Table 3).

**Table 3:** Correlation coefficients of MYO‐band metrics with EDSS, self‐reported EDSS, and the quality of life WHODAS questionnaire.

Assessment	Combined right and left limbs	Dominant limb measure	Nondominant limb measure
Finger taps			
Time‐based analysis			
EDSS	0.51	0.43	0.42
Self‐EDSS	0.49	0.48	0.42
WHODAS	0.41	0.37	0.34
Textural analysis			
EDSS	0.77	0.73	0.68
Self‐EDSS	0.80	0.72	0.73
WHODAS	0.82	0.76	0.76
Foot taps			
Time‐based analysis			
EDSS	0.65	0.53	0.56
Self‐EDSS	0.61	0.54	0.53
WHODAS	0.36	0.29	0.39
Textural analysis			
EDSS	0.82	0.73	0.76
Self‐EDSS	0.81	0.72	0.78
WHODAS	0.82	0.77	0.76

For all correlation coefficients (*r*_s_ – Spearman correlation coefficient) in the table, the *P*‐value was < 0.0001. Correlations are for the replication data (not from the data used to create the models).

In multivariable regression analyses adjusting for age and sex, for every additional second to complete a tapping task, there was an associated 0.21 higher EDSS score for combined upper limbs, and 0.25 higher EDSS score for combined lower extremities. Similar results were seen for patient‐reported EDSS and WHODAS (Table 4).

**Table 4:** Multivariable regression models of the association of MYO‐band metrics with EDSS, self‐reported EDSS, and the quality of life WHODAS questionnaire.

Assessment	Combined right and left limbs	Dominant limb measure	Nondominant limb measure
	*β* (95% CI)	*β* (95% CI)	*β* (95% CI)
Finger taps			
Time‐based analysis			
EDSS	0.21 (0.059, 0.36)	0.22 (0.031, 0.31)	0.25 (0.015, 0.49)
Self‐EDSS	0.21 (0.049, 0.37	0.27 (0.074, 0.47)	0.26 (0.025, 0.50)
WHODAS	1.45 (0.28, 2.67)	1.60 (0.11, 3.09)	1.68 (−0.14, 3.5)*
Textural analysis**			
EDSS	0.90 (0.63, 1.17)	6.08 (4.02, 8.18)	6.02 (3.77, 8.27)
Self‐EDSS	0.93 (0.66, 1.20)	6.68 (4.43, 8.93)	7.56 (4.9, 10.3)
WHODAS	0.98 (0.74, 1.22)	48.9 (35.1 ,62.7)	46.6 (32.9, 60.3)
Foot taps			
Time‐based analysis			
EDSS	0.25 (0.12, 0.38)	0.25 (0.095, 0.40)	0.29 (0.11, 0.47)
Self‐EDSS	0.27 (0.13, 0.41)	0.22 (0.051, 0.39)	0.30 (0.10, 0.50)
WHODAS	1.21 (0.014, 2.41)	1.12 (−0.27, 2.59)*	1.73 (0.18, 3.28)
Textural analysis**			
EDSS	0.93 (0.68, 1.18)	5.97 (3.74, 8.2)	5.86 (3.93, 7.78)
Self‐EDSS	0.92 (0.68, 1.16)	6.52 (4.21, 8.83)	7.49 (5.37, 9.61)
WHODAS	0.98 (0.74, 1.22)	44.1 (31.7, 56.5)	47.6 (33.4, 61.8)

The beta coefficient for a unit change in the MYO‐band metric predictor is presented for each model/outcome along with the 95% CIs. These results are for the replication data (not data used to derive models). The *P*‐values for time‐based analyses were all less than 1 × 10^−4^ except *nondominant upper limb association and dominant lower limb with WHODAS *P* > 0.05. *P*‐values for textural analyses were all less than 1 × 10^−10^. **Due to the nature of and the varying units of the different time and textural predictor variables the magnitude of the coefficients is not directly comparable across all of the statistical approaches. *P*‐values for the textural analysis fusion metrics were all lower than for single limb measures (<1 × 10^−27^ vs. <1 × 10^−18^).

### Texture‐based analyses

All three sensors (ACC, GYR, and sEMG) were used to generate textural features of tap performance in all limbs. Waveform textural feature extraction was performed as described in the Methods and the Supplemental Appendices. Overall, the extracted textural features demonstrated greater tap performance irregularity across 20 tap segments in those with higher versus lower EDSS scores. The strength of the approach is capturing this irregularity in high resolution, mathematical detail.

To understand which of the textural features were more likely to be describing phenomena of interest (limb disability) versus fluctuation related to noise, we applied a partial least squares (PLS) based‐optimization method followed by statistical analysis to all of the textural features for all three sensors to derive a single summary textural feature value (“T‐metric”) for each participant with MS (60% of data used for training). This T‐metric single scalar value was then modeled as a predictor of physician‐ and patient‐reported EDSS as well as WHODAS scores (in the remaining 40% of data—the replication dataset, Tables 2, 3, 4). As an example, for foot taps, an informative combination of features is presented in Supplemental Figure 2, demonstrating a heatmap of the PLS textural feature fusion, the final feature marker from the PLS method (T‐metric) and its association with physician EDSS.

We evaluated performance of the PLS‐derived final T‐metric by computing the ICC to quantify repeatability (Tables 2). The ICCs were all in the good to excellent range for the final PLS statistics (Table 2). There were strong Spearman correlations between the T‐metric value and physician‐ and patient‐derived EDSS and WHODAS as shown in Table 3.

Multivariable regression analysis adjusted for age and sex demonstrated that the T‐metric was strongly associated with physician‐ and patient‐reported EDSS as well as WHODAS (Table 4). Adjustment for sex did not change model results and while age appeared to modestly change the results (10% or less), the impact of age on textural features was notably less than for time‐based analyses. For every 1 unit increase in the PLS T‐metric for dominant hand finger taps, there was a 6.08 unit higher EDSS score, and for every 1 unit increase in the final texture‐based PLS‐derived metric for the dominant foot taps, there was a 5.97 unit higher EDSS. Similar results were obtained for the patient‐reported EDSS and WHODAS as shown in Table 4. The magnitudes of these effect sizes presented in the table are large due to the unit size of the PLS‐derived T‐metric. This is simply related to the numerical calculation of the T‐metric and the units are arbitrary or “unit‐less.” This is compared to the units of seconds for the time‐based metrics. For a clinically relevant smaller difference in the PLS‐derived T‐metric, such as a 0.2 change, the difference in physician EDSS, as an example, would be 1.2.

## Discussion

With great future promise as a new neurological vital sign and for applications beyond MS, we have demonstrated that spatiotemporal extracted features from a multi‐sensor device are reliable and easy to use in practice, and can distinguish differences in limb function. Both time and textural analyses of sensor data distinguish cases from controls and have strong associations with physician‐ and patient‐reported disability. Textural features using data merged from three different sensors had the best correlation with the gold standard EDSS and were less affected by patient age than time‐derived features. Notably, these features were also as strongly associated with patient‐reported outcomes. As the testing protocol with the MYO takes only a few minutes compared to the half hour for an EDSS and could be administered by research staff or medical assistants in the clinic or potentially by patients in their own homes, this testing paradigm has broad implications for future trials and clinical monitoring in MS. This multi‐sensor device and analytical approach could also be applied to other neurological or traumatic injuries. Future applications could include stoke, Parkinson’s disease, or amyotrophic lateral sclerosis (ALS).

Neurological patients receive vital sign measurements of blood pressure and heart rate with every clinic visit. While these data give a snapshot of cardiovascular health, there historically has been no equivalent for neurological function. One could easily argue that development of a neurological vital sign would have more direct import for neurological patients than the cardiovascular measures. In the same time it takes to measure blood pressure and heart rate, we were able to use a single device to provide a discrete numerical estimate of both upper and lower limb function. The MYO‐band is smaller than a blood pressure cuff and comfortable. Testing can be administered by nonclinical personnel and could even be used remotely by patients for telemedicine visits in the future.

In addition to the benefits of this new vital sign for clinical care, there is a high demand for improved outcome measures in MS for clinical trials.^10^ The limitations of the gold standard EDSS exam have been well described elsewhere^11^, ^12^ but include interrater variability and insensitivity to change over the short term. The strong dependence of the scale on ambulatory status means for a patient using a cane (EDSS 6.0) even if she had worsening of her dominant upper limb function, her official outcome in a trial would be “stable” as long as she continued to use a cane and did not start using a walker (EDSS 6.5). A device and algorithm as reported here, which gives equal importance to the upper limbs and decreases interrater variability, may allow much shorter trial durations to detect a treatment effect.

This type of biosensor approach also offers potential advantages over components of the MS Functional Composite (MSFC). In the MSFC, the nine‐hole peg test (9HPT) is used to assess upper limb function and the timed 25‐foot walk (T25FW) test evaluates lower limb function. These tests can be very useful in tracking progression, but use different scales of measurement for upper and lower limbs. With the MYO‐band approach, we applied the same device and analytics for upper and lower limbs; thus, the results are comparable. One can measure progression similarly in any limb. Comparing the MYO‐band to the T25FW, each leg’s performance is measured rather than a composite of walking function. With the band device we are better able to evaluate subtle changes within one leg. In this proof of concept study we were able to demonstrate differences in foot tapping function even in those with normal ambulatory function.

The highest reproducibility of the MYO‐band measures for the upper limbs was for the dominant side, which is intuitive given importance of handedness for upper limb function. For textural analyses the strongest correlations with gold standard EDSS was with the fused statistic for right and left limbs.

One of the advantages of the MYO multi‐sensor system is inclusion of surface EMG. This sensor has not been commonly used in MS wearable studies. While sEMG has been studied in the past as a possible diagnostic tool for neuromuscular disorders,^13^ it is important to note that in our algorithm we are not trying to establish the surface EMG as a replacement for standard needle EMG. Rather we have used it as an additional sensor to provide precise timing of motor tasks and estimates of irregularity in movement and to complement accelerometer and gyroscope data.

Tapping tasks were selected for analysis for simplicity of the motion, mathematical advantages in analyzing repeated movements, and ability to use the same software for upper and lower limbs. Instructions to the patient are simple (and could be given by nonclinical staff) and additional equipment is not required in the clinic. Another advantage is the eventual generalizability of the software to other neurological diseases for which tapping movements are used in diagnosis and follow‐up of patients (Parkinson’s, ALS, and stroke).

Regarding scalability and the long‐term utility of this algorithm, while the current work was proof of concept, it is our vision that a multi‐sensor device (these types of sensors are now widely available at low cost) and software with a commercial grade user interface that could run on any PC would be accessible to practices and eventually patients for home measurements. Both acquisition and analytical software would be available without need for any local signal processing expertise. After additional study to demonstrate sensitivity to changes over time and reproducibility within a multisite setting, there are no obvious limits to future scalability.

Limitations of the current work include cross‐sectional design, and analyses are already underway to address sensitivity of the textural feature algorithm to detect within patient change over time in a real‐world clinical setting. While we anticipate this method to be more sensitive than the EDSS to deficits and change over time, in the absence of a better ground truth or gold standard, statistical approaches to prove superiority for a novel technology are challenging. Our inclusion of patient‐reported outcomes adds validity to our approach.

Other strengths of our work include the sophisticated image processing approach to extracting features from the raw signal data and creation of a simplified final statistic to represent the complexity and patterns of irregularity in the data. We had consistent results across physician and two different patient‐reported outcomes. We demonstrated good to excellent intertest reliability and rigorously evaluated which metrics extracted from the sensor data performed better than others. The examinations took place in routine clinical practice and MYO tests took only a few minutes. The bracelet fits on a wide variety of body types on both upper and lower limbs. This work also demonstrates that commercially available sensors can be validated for clinical use at low development cost.

This device and analytical approach holds potential as a new neurological “vital sign” that could be checked by nonclinical personnel in routine visits. Future validation of this multi‐sensor algorithm will include longitudinal study in MS patients and both cross‐sectional and longitudinal studies in other neurological diseases.

## Conflict of Interest

Alireza Akhbardeh has no disclosures. Jennifer Arjona has no disclosures. Kristen Krysko is supported by the Sylvia Lawry Physician Fellowship from the National MS Society (FP‐1605‐08753 (Krysko)) and a Biogen Fellowship grant. Bardia Nourbakhsh has current grant support from PCORI. P.A. Gourraud is supported by the ATIP‐Avenir INSERM program and the Region Pays de Loire ConnecTalent, ARSEP Foundation (France), and the Nantes University Foundation. Jennifer Graves has received recent grant and clinical trial support from the National MS Society, Race to Erase MS, UCSF CTSI RAP program, Biogen, and Genentech. She has received honoraria from Biogen, Novartis and Genzyme for education events.

## Author Contributions

Alireza Akhbardeh contributed to signal processing and statistical analysis plan, performed the signal processing and parts of the statistical analyses, and edited the manuscript. Jennifer Arjona managed the data collection for the project, contributed to the analyses and edited the manuscript. Kristen Krysko provided feedback on project design and data interpretation and edited the manuscript. Bardia Nourbakhsh contributed patient data, provided feedback on project design and edited the manuscript. P.A. Gourraud contributed to study design and edited the manuscript. Jennifer Graves designed the study, provided participants for the study and performed study evaluations, created the signal processing and statistical analysis plan, and drafted and edited the manuscript.

## Supporting information


**Data S1.** Methods Supplemental Appendices.
