# Supplementary material for: Novel MS vital sign: multi‐sensor captures upper and lower limb dysfunction
Source: Ann Clin Transl Neurol. 2020 Feb 26;7(3):288–95. doi: 10.1002/acn3.50988 (PMC7085995; doi:10.1002/acn3.50988)
Supplement: Supplementary file 1 — Data S1. Methods Supplemental Appendices. [file ACN3-7-288-s001.docx]

**Methods Supplemental Appendix A: Textural Analysis**

To quantify each tapping event from different sensor data (surface EMG, accelerometer, and gyroscope) we developed an algorithm based on extracting Haralick features and statistical measures. This algorithm is used to extract the following features: ‘mean value for each tap’, 'overall_energy', 'overall_entropy', 'haralick_energy', 'haralick_correlation', 'haralick_contrast', 'haralick_homogeneity'. To calculate these features, we first form a matrix using different channels of sensor data. For example, for the accelerometer we have 3 channels (X, Y, and Z). By stacking these channels, we get so-called tap event matrix P. We then calculate co-occurrence matrix for different features by use of the formulas in Supplemental Table 1.

**Supplemental Table 1: Definition of Haralick features for textural analysis**

| Feature Name | Definition | Formula |
| --- | --- | --- |
| Haralick-Contrast | A measure of the intensity contrast between a sample and its neighbor over the whole matrix P. Contrast value is 0 for constant samples.  Range = [0 (size(matrix,1)-1) ^2^] | $\sum_{i,j} \left\vert i-j \right\vert^{2}*P(i,j)$ |
| Haralick-Correlation | Measures how correlated a sample is to its neighbor over the whole matrix P. Range = [-1 1] | - |
| Haralick-Energy | Measures the sample’s energy, which is the sum of squared elements in the matrix P. Range = [0 1] | $\sum_{i,j} {P(i,j)}^{2}$ |
| Haralick Homogeneity | Measures the closeness of the distribution of elements in matrix P | $\frac{\sum_{i,j} P(i,j)}{1-\vert i-j\vert}$ |

To have a complete evaluation of cross-channel relationships for each sensor, we calculate Haralick features for different orientations as shown in supplemental Figure 1. We then calculate the average of textural features in the 4 orientations to get one value for each feature in each tap. By aggregating extracted textural features across taps we get an array of features for the 20 taps. If we stack up the features array across subjects we will have a matrix for each textural feature.

**Supplemental Figure 1: Signal processing for extraction of textural-based features**

*Haralick features are calculated for 4 different orientations as below*

**Methods Supplemental Appendix B: PLS optimization and derivation of final T-metric**

The input to PLS based optimization is aggregated sensor/textural-features combinations:

X=[X_EMG_, X_Accelerometer_, X_Gyroscope_], where X_EMG_, X_Accelerometer_, X_Gyroscope_ are different combinations of normalized textural features.

As we employed 14 channels of data from 3 sensors (sEMG, accelerometer and gyroscope) and 7 features (‘mean value for each tap’, 'overall_energy', 'overall_entropy', 'haralick_energy', 'haralick_correlation', 'haralick_contrast', 'haralick_homogeneity'), there were 35 possible combinations of features (3 features in each combination) and 7 sensor combinations. In the PLS optimization process we had 35x7=245 iterations. In each iteration, PLS reduces features/sensor combination array X to N dimensions (elements). We tested different values for N to find the optimum value. The ground truth (physician or patient-reported EDSS or WHODAS) was used to find the most important and optimal combinations. Sixty percent of the data was used for PLS model training. PLS signal is defined as PLS variation over time. Standard statistical methods (mean, standard deviation, skewness, kurtosis) are applied to the PLS signal to create a single summary statistic, a so-called “T metric,” representing the textural features from the MYO for each limb in each participant.

The formula to calculate T-metric is as following:, where i is participant index, is PLS signal, t means time and f(.) means statistical measure (mean, std, etc.). By stacking we get H matrix. H=[;…. Each row in H is the PLS signal for a participant. The matrix H has the size of MxN, where M is number of participants and N is number of samples for PLS signal over time. First subplot (A) in Supplemental Figure 2 shows matrix H. Second subplot (B) shows the final marker or “T-metric” value for each participant as a bar plot. The third bar plot (C) shows the physician reported EDSS for each participant.

**Supplemental Figure 2: Partial least squares best combination of textural features from foot taps associated with physician reported EDSS.**

A B C

Fusion results for the final selected textural features from all 3 sensors from partial least squares analysis. A) Heatmap of final textural features fusion, where color code changes from blue (lower values) to yellow (higher values). Values are in au (arbitrary unit). B) Final PLS-derived statistical feature ordered by patient’s EDSS Score C) EDSS score. The final textural feature value (“T-metric”) was associated with physician-reported EDSS. An EDSS of -1 indicates a healthy control.
